# Characteristics of Entrained Air Voids in Hardened Concrete with the Method of Digital Image Analysis Coupled with Schwartz-Saltykov Conversion

**DOI:** 10.3390/ma14092439

**Published:** 2021-05-08

**Authors:** Teemu Ojala, Yanjuan Chen, Jouni Punkki, Fahim Al-Neshawy

**Affiliations:** Department of Civil Engineering, Aalto University, 02150 Espoo, Finland; teemu.ojala@aalto.fi (T.O.); jouni.punkki@aalto.fi (J.P.); fahim.al-neshawy@aalto.fi (F.A.-N.)

**Keywords:** digital image analysis, air-void system, air content, stereology, Schwartz-Saltykov method, void distribution

## Abstract

This paper presents the characteristics of air void systems in hardened concrete with the method of digital image analysis (DIA) coupled with Schwartz-Saltykov (SS) conversion. The results indicate that the DIA method coupled with SS conversion estimates the air content with more accuracy than it would without SS conversion; the correlation between air content obtained from the DIA method, and that from the thin section (TS) method is as good as the correlation observed between the pressure saturation (PS) method and the TS method. It was also found that the DIA method shows a better correlation with the TS method when the spacing factor without SS conversion is considered, while both methods show poor correlations when the corresponding specific surface is considered. In addition, it indicates that the peak of three-dimensional size distribution (3-DSD) of air voids after SS conversion falls in smaller voids, and 3-DSD of air voids shifts to a narrow size range, in comparison with the 2-DSD without SS conversion; the shape of the 3-DSD air voids remains constant irrespective of the class widths. Increasing the number of classes can minimise the standard deviation in the estimation, however, it also results in a leap in voids volume density, which will influence the estimation of air content.

## 1. Introduction

Since 1930, air-entrained concrete has been introduced to improve the resistance to freeze-thaw, and in 1954, various parameters were proposed by Powers to characterise the air void system in concrete [1]. Since then, many studies have appeared to describe the methods and calculate the air void parameters for the purpose of determining concrete resistance to freeze–thaw [2,3,4,5,6,7,8,9]. Some methods have been mathematically and statistically combined with the science of stereology [10,11,12]. Generally, there are three stereological techniques most used, i.e., the point count, the lineal analysis, and the section analysis (areal analysis), varying in terms of accuracy and ease of use. The most utilised methods for air voids analysis in concrete are the Chayes point method and the Rosiwal linear traverse method, which have been standardised by ASTM C457 [13], as well as EN 480-11. These techniques involve optical microscopic (OM) techniques and stereology science. However, these traditional standards have inevitable drawbacks, i.e., tedious manual measurements, time-consuming, and operator dependent. Image analysis has been very popular in air voids analysis since the 1970s due to its availability, simplicity, and low cost [14,15,16,17,18]. For instance, Dewey et al., in 1991 [19], applied the image analysis technique to characterise the air void system in hardened concrete. This study demonstrated that image analysis provided a viable alternative to traditional lineal traverse and modified point count methods for characterisation of air void systems in hardened concrete; however, the lineal analysis consistently provided a higher value of total specific surface than areal analysis, despite that both methods provided a similar estimate of total air content. Thus, further investigation should be made with respect to which technique (lineal or areal) is more appropriate. Although it is possible to use linear intercepts to estimate volume size distribution, it is believed that methods based on profile diameters or areas provide better results [20].

In recent years, the technique of image analysis has evolved very rapidly thanks to progress in image acquisition, and the development of algorithms and software for general or specific applications. Various semiautomatic and fully automatic devices have been developed and are currently available for rapid data collection and analysis. Especially, research on air voids analysis has been transferred to obtain two-dimensional (2D) information about diameter, section area, and perimeter using digital image analysis (DIA), because this can avoid the drawbacks of traditional standards, and it has the merits of availability, simplicity, and low cost. Most articles that have been published about air voids parameters (such as air content, specific surface, spacing factor, and air voids distribution) measurement by DIA methods including the elaboration of DIA testing procedures [11,21,22,23]. However, less attention has been focused on accurate estimation of DIA measurements. Moreover, concerning DIA measurements, there is still much more work to do, e.g., algorithm development of the conversion of results from 2D to 3D (develop stereological methods), making the 2D measurements comparable with the actual distribution, and the verification/comparison of the DIA’s measurement and traditional measurements.

In this study, the DIA method coupled with a stereology mathematical algorithm are adopted to characterise the air-entrained voids. Traditional parameters about air voids: total air content, specific surface, and the powers spacing factor, are computed. Volume distribution of air voids and areal distribution of air voids are shown, separately. A comparative study on the air voids information between this DIA method and the thin section method, and the pressure saturation method, has been presented in detail.

## 2. Algorithm of Conversion from 2D to 3D

Stereology is defined as the body of methods for the investigation of 3D space when only 2D sections through solid bodies, or their projections on a surface, are available. Thus, stereology is also an extrapolation from two to three dimensions [24]. Stereological methods have been theoretically developed in all kinds of particles or voids (with the shape of spheres, ellipsoids, convex particles, lamellar spacing), especially in the case of spherical particles due to their simple shape and maximum symmetry. Entrained air voids are such ideal spheres, and most stereological mentions in the literature are related to concrete science. One of the first principles of stereology based on a homogeneous dispersed phase, area fraction, lineal fraction, and point fraction, is a statistically unbiassed estimated for the volume fraction.

(1) $$P_{P}=L_{L}=A_{A}=V_{v}$$

This principle provides the basis for the Chayes point method and the Rosiwal linear traverse method of air voids measurements in concrete, as well as the base-ground for the conversion of the mathematical algorithm from 2D to 3D.

Several fundamental theoretical relations in stereology utilise the principles of mathematical probability, and equations have been derived relating the surface parameters to the actual parameters in the solid. Several 2D to 3D conversion solutions have been adopted to determine the air void distribution from the distribution of the sectional area [12,23,25]. A conversional solution introduced by Wicksell in 1925 is the back-substitution method, advanced by Scheil and Schwartz, and later modified by Saltykov [26,27,28,29,30]. The developed Schwartz-Saltykov method (SS method for short) can derive the 3D pore distribution from the 2D histogram without assumptions about the continuous function for size distribution of particles [31]. Thus, the SS method is considered superior to earlier approaches and is widely used [11,23,32]. In 2003, Takahashi [33] evaluated the accuracy of 3-DSD with estimations from the SS method and concluded that the quantitative 3-DSD can be obtained from the reasonably estimated values of the shape coefficient and the correctly determined relative frequency. The SS method is one of the most used stereological approaches to obtain the size distribution of the secondary phases in a unit volume of material, starting from a distribution of particle diameters in two dimensions [32]. SS conversion was adopted as the stereology mathematical algorithm that calculates the air voids’ 3D geometric properties from 2D measurements in this research.

Air voids in hardened concrete can be entrained and entrapped. Generally, the deliberately entrained air voids are characterised as discrete, individual voids of spherical shape, which are uniformly distributed throughout the cement matrix, and are not interconnected with each other. The size of entrained air voids ranges from 0.01 mm to 1 mm according to ASTM C125, and ranges between 0.02 mm and 0.5 mm have been reported in the literature [24]. The accidently entrapped air voids range up to several millimetres, much bigger than entrained air voids, with random size and shapes which are irregular and non-spherical. Entrapped air voids with a size above 1 mm have been defined according to ASTM. However, ASTM C457 states that there is no provision to distinguish between entrapped air voids and entrained air voids. In this research, the entrained air void was set as a void in which the diameter should not exceed 0.8 mm; thereby, the air content was defined as all the entrained voids no more than 0.8 mm in equivalent diameter that can be detected with the DIA method, and the entrapped air voids were not counted.

According to the stereology mathematical algorithm of SS conversion, the following assumptions are made:

1. Air voids in the system are spherical, and the pores on the test disc are circular.
2. Voids are distributed randomly throughout the specimen without any regular packing.
3. The size of voids is measurable even if they overlap on the image(plane) or space.

In view of the characteristics of air voids mentioned above, it is obvious that entrained air voids meet the requirements of the assumptions, but entrapped air voids do not. Thus, the statistic of the entrained air voids is a suitable case for SS conversion application, even without considering the form factor [34] (the form factor will be equal to 1 for a perfect circle and will decrease as the perimeter of the measured section becomes more irregular) due to the entrained air voids being round enough. Thus, the DIA method combined with the SS conversion are intended to offer the information about entrained air voids.

In SS conversion, to avoid confusion, the measured 2D voids are called “section”, while the generated 3D voids are called “sphere”. The measured 2D sections are split into *k* classes of width Δ, which is defined as:

(2) $$\Delta =d_{A,max}/k$$

where $d_{A,max}$ refers to the maximum equivalent diameter of the measured sections, and *k* refers to the number of classes. The voids within each class are considered to have the same diameter. A section with a certain size (e.g., in class *i*) appears as a result of the interception of a void having a size equal to or greater than the section size. Therefore, when counting the number of voids per unit volume in a certain class *j* from the number of voids in the unit area, it is necessary to subtract the number of voids in the bigger classes multiplied by the probability that they have generated a section in class *j*. The general formula of the SS conversion using the successive subtraction process is expressed as [33]:

(3) $$N_{V}\left(j\right)=\frac{1}{\Delta }\left(\alpha \left(j,j\right)\right)N_{A}\left(i\right)-\alpha \left(j+1,j\right)N_{A}\left(j+1\right)-\cdots -\alpha \left(k,j\right)N_{A}\left(k\right)\;for\;i\geq j$$

where $N_{V}\left(j\right)$ refers to the number of voids in class j per unit volume of the sample, and $N_{A}\left(i\right)$ is the number of sections in class i per unit area of the intersected plane of the sample. The values of i and j are up to maximum value of k. Saltykov originally solved the coefficients in the formula using the maximum number of classes of 15. Still, the coefficients can be generalised to any desired number of classes, and computation can be performed rapidly on a computer. The values of coefficient α(i,j) are generalised by the following procedure:

(4) $$\alpha \left(j,i\right)=\{\begin{array}{c} \;\;\;\;\;\;T\left(i,i\right),\;i=j\\ -\overset{j-1}{\underset{m=i}{\sum }}\alpha \left(m,i\right)T\left(m,j\right),\;i<j \end{array}$$

where T(i,j) is the translation coefficient, defined as:

(5) $$T\left(i,j\right)=\{\begin{array}{c} \frac{1}{A\left(j,j\right)},\;i=j\\ \frac{A\left(i,j\right)}{A\left(j,j\right)},\;i<j \end{array}$$

The shape factor A(i,j) is determined by the successive substitution of integer values of i and j, and defined as below:

(6) $$A\left(i,j\right)={\left(j^{2}-{\left(i-1\right)}^{2}\right)}^{1/2}-{\left(j^{2}-i^{2}\right)}^{1/2},\;i\leq j$$

The procedure of conversion from 2D to 3D consists of the following four steps.

1. Predetermination of the maximum diameter of air void, and the width of classes Δ, thereby determining the maximum class number of k;
2. Determination of 2D conditions: $N_{A}$ the number of sections per unit test area ($mm^{-2}$); the coding that computed $N_{A}$ and $N_{V}$ was written in MATLAB R2020a based on the above formulae;
3. Obtain $N_{V}$ the number of spheres per unit volume ($mm^{-3}$);
4. Distribution histograms $N_{A}\left(i\right)-d_{A}\left(i\right)$ and $N_{V}\left(j\right)-d_{V}\left(j\right)$ are generated and denoted as 2-DSD and 3-DSD for short.

Furthermore, the other histograms from the same data sections can be obtained by repeating the steps 2 and 3 by changing the Δ values.

**Figure 1:**
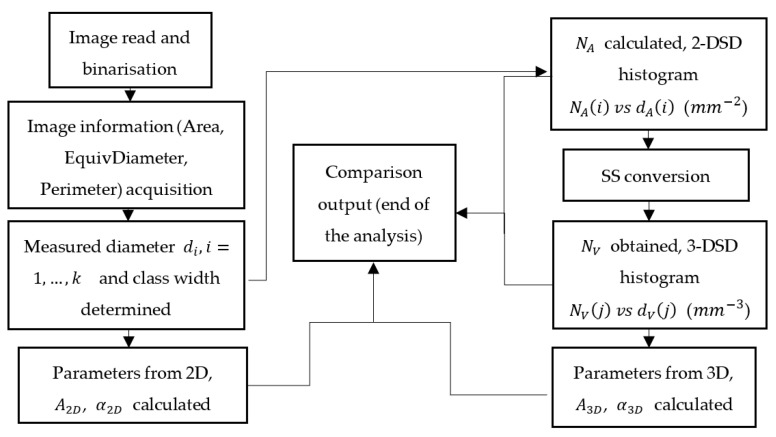
Simplified logical procedure of analysis in MATLAB.

A computing MATLAB procedure is depicted in Figure 1 regarding the 2-DSD, 3-DSD, and parameters related to the air void structure. The involved parameters in this figure will be introduced in detail in the next section.

## 3. Parameters of the Air Void System

***Total air content*** is the proportion of the total volume of air voids to the total volume of concrete, including all the constituents of the concrete and total volume of air. Given that air voids contribute to the frost resistance of the hardened cement paste only, it is also useful to express the air content as a percentage of the cement paste volume, i.e., volume of cementitious materials, water, and air.

***The specific surface*** is usually defined as the total surface area of the air voids divided by their total volume. ASTM C457 mentions that for concrete to be durable to freezing and thawing effects, the specific surface of air voids in concrete should be 23.6 and 43.3 mm^−1^, respectively [13]. High values of specific surface imply a fine air void system. Therefore, for air void systems that have similar total air contents, the specific surface can be a useful indicator of the air void distribution, despite that the specific surface cannot provide any information about the actual number of air voids having a specific size.

***The spacing factor*** has been the best indicator that the air void system can resist a frost attack. It was first proposed by Power [35], which was the basis for ASTM C457 spacing factor ($\bar{L}$). In 1954, Powers proposed a simple model to determine the spacing factor, which be calculated from air content, paste content, and specific surface of air voids by one of the two formulae in Equation (7):

(7) $$\bar{L}=\frac{3}{\alpha }\left[1.4{\left(\frac{p_{cem}}{A_{a}}+1\right)}^{1/3}-1\right]\;if\;\frac{p_{cem}}{A_{a}}\geq 4.342$$

(8) $$\bar{L}=\frac{p_{cem}}{\alpha A_{a}}\;if\;\frac{p_{cem}}{A_{a}}<4.342$$

where $\bar{L}$ is the spacing factor (mm), $p_{cem}$ is the cement paste content (%), $A_{a}$ is the total air content (%), and α is the specific surface of air voids mm^−1^. It should be emphasised that when calculating the spacing factor of an air void system, the air content must be expressed as a fraction of the air-free paste content. In general, in air-entrained concrete, the ratio of $p_{cem}/A_{a}$ is greater than 4.342, hence, Equation (7) is used in most cases. ASTM C457 requires that the spacing factor is between 0.10 and 0.20 mm to ensure the production of concrete durable to freezing and thawing. However, two concretes with the same spacing factor will probably show different resistance to frost attack. In addition, the spacing factor is not an independent parameter, and it results from a calculation with the paste content, the air content, and specific surface input, therefore, the spacing factor will not be treated further in this research.

### 3.1. Parameters of Air Void System Obtained from Area Measurement (2D Parameters)

In this research, all involved parameters of the air void system from the DIA method coupled with SS conversion were defined as 3D parameters, while these parameters from the DIA method without SS conversion are defined as 2D parameters.

#### 3.1.1. Air Content

The parameters are obtained from the area measurement with the DIA method. As for a representative 2D plane of concrete, assuming the air content is equal to the area percentage of air voids (as identified in the binary image) [24], and expressed as the ratio of sum of air void surface area to the surface area of the specimen, Molendowska et al., in 2020, gave the expression as [11]:

(9) $$A_{2D}=\frac{\sum^{\;}Area}{AOM}$$

where $A_{2D}$ represents the air content from the area measurement, Area is the surface area of identified sections/voids on the plane, and AOM is the surface area of measured plane. It should be noted that $A_{2D}$ is not a stereological value. Regardless of if the expression is reasonable, the purpose of this study is to compare the results with those after SS conversion.

#### 3.1.2. Specific Surface of Air Voids

In 1949, Willis demonstrated that the total volume of air voids and their total specific surface can be estimated from the mean air-void intercept or chord length obtained from a linear traverse [35]. Assuming all voids to be spherical and using the geometric probability concept, Willis showed that:

(10) $$\alpha =\frac{4}{\bar{l}}$$

where α is the total specific surface, and $\bar{l}$ is the average chord length. This equation was adopted in ASTM C457 to determine α, with methods of linear traverse or point-count. Based on the same stereology concept, in 1991 Dewey [19] first mentioned that Willis (1951) developed an expression for the total specific surface from an area analysis such as $\alpha =\left(16/\pi \right)\left(\sum^{\;}n_{i}d_{i}/\sum^{\;}n_{i}d_{i}{}^{2}\right)$, where $d_{i}$ is the equivalent diameter of the objects,$\;n_{i}$ is the number of objects in the i_th class. Pleau et al. [21] also gave a similar expression to calculate the specific surface with image analysis. During digital image processing, the specific surface of objects can be determined by computing the perimeter of those objects on a representative 2D plane, because the area, perimeter, and equivalent diameter of objects can be computed by counting pixels, which can be straightforward. Hence, the expression of total specific surface mentioned above can be derived as:

(11) $$\alpha_{2D}=\frac{4}{\pi }\frac{\sum^{\;}Peri}{\sum^{\;}Area}$$

where $\alpha_{2D}$ is denoted as the specific surface from area measurement, and Peri (mm) and Area (mm^2^) are the perimeter and area of sections in the 2D plane, respectively. It should be noted that $\alpha_{2D}$ is also a stereological value, as the α in ASTM C457, but it was calculated with the parameters from area measurement without SS conversion, thus, it was sorted as a 2D parameter in this research.

### 3.2. Parameters of Air Void System after SS Conversion (3D Parameters)

#### 3.2.1. Air Content after SS Conversion

According to the definition of total air content mentioned above in Section 3, the air content can be expressed as the volume fraction of air voids. Since the SS conversion supposes that air voids are randomly dispersed, the estimated total volume fraction can be calculated as follows:

(12) $$A_{3D}=\frac{\pi }{6}\overset{k}{\underset{i=1}{\sum }}N_{V}\left(i\right)d{\left(i\right)}^{3}$$

where $N_{V}\left(i\right)$ is the number of voids per unit volume of the sample, and d(i) the mean pore diameter in the i_th class. This expression of volume fraction can also be used to calculate volume porosity in the high burnup structure [32,36,37].

#### 3.2.2. Specific Surface of Air Voids after SS Conversion

From the definition of specific surface of air voids in Section 3, it is a calculated parameter representing the total surface of all air voids divided by the total volume of voids, in mm^−1^. It can be derived through SS conversion as below:

(13) $$\alpha_{3D}=\frac{\sum_{i=1}^{k}N_{v}\left(i\right)\pi d{\left(i\right)}^{2}}{\sum_{i=1}^{k}N_{v}\left(i\right)\frac{\pi }{6}d{\left(i\right)}^{3}}$$

(14) $$\alpha_{3D}=\frac{\pi \sum_{i=1}^{k}N_{v}\left(i\right)d{\left(i\right)}^{2}}{A_{3D}}$$

The similar expression of specific surface of air voids was found in Elsen et al.’s work in 1994 [24] and Snyder’s work in 1998 [38].

## 4. Materials and Experimental Methods

### 4.1. Materials and Sampling

Three mixtures of concrete, denoted GV01, GV10, and GV14, were investigated in this study. The first two mixtures GV01 and GV10 were made with cement Plus CEMII/B-M(S-LL) 42.5N, and the third mixture was made as sulphide-resistant concrete using cement SR CEMI 42.5N. Both the cements were manufactured by Finnsementti Oy in Parainen, Finland. For each mixture, two specimens were cast using vibration times of 15 and 60 s, thereby, six concrete specimens (500 × 600 × 250 mm^3^) were mixed and cast on a ready-mix station. Each of the mixtures presented different concrete qualities that are often used in Finland in infrastructures where concretes are exposed to freeze and thaw cycles and salts. The concrete quality and composition of the cast mixtures are shown in Table 1. All the concretes were super-plasticised with MGL Glenium Sky 600 (AEA for short), and air entrained with MasterAir 100, manufactured by BASF Oy in Helsinki, Finland.

**Figure 2:**
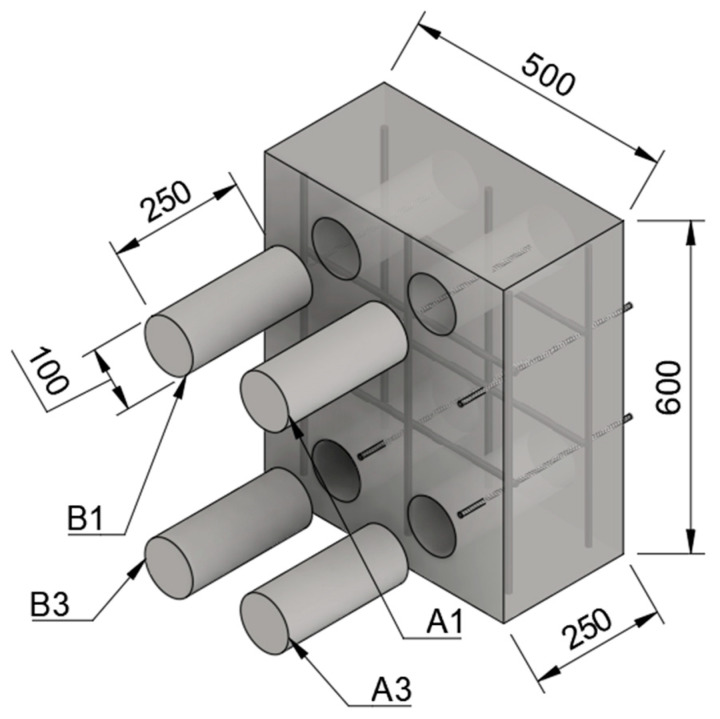
An illustration of the specimen (500 × 600 × 250 mm^3^) where the drilled cores A1, B1, A3, and B3 are represented.

For each concrete specimen, two parallel core samples with a diameter of 100 mm, were drilled from top (A1 and B1) and bottom (A3 and B3), respectively, as depicted in Figure 2. Afterwards, the four samples were cut from the parallel cores and prepared for the parallel testing of TS, PS, DIA and compressive strength test. The code of samples was denoted as GV01_15_1, the first part is the mixture of concrete, the second part means the duration of vibration time, and the third part represents the location of the sample where it was retrieved (1 and 3 represent the top and bottom, respectively).

### 4.2. Experimental Methods

#### 4.2.1. Thin Section Method

Thin-section specimens were analysed using a guideline called VTT TEST-R003-00-2010. In the TS method, entrained air pores are defined as voids that have a longest diameter between 0.020 and 0.800 mm. Two sections/planes were analysed from each core, and the results represent the mean of these two sections. A total area of approximately 3500 mm^2^ was inspected (requirement over 3000 mm^2^) and the amount of analysis points were 1783 ± 16 (requirement over 1500). The distance between the analysis points was 1.0 mm.

#### 4.2.2. Pressure Saturation Method

For the PS method, three parallel discs with the diameter of 100 mm and thickness of 20 ± 2 mm were sawn from the cores. To carry out the PS test, samples must go through the following steps. First, the samples were saturated in water with normal pressure and room temperature of 20 ± 2 °C, so that they were half submerged for 24 h, and fully submerged under water for another 48 h. After the samples were fully saturated, the weights of the samples in air and under water were documented as $W_{sat}$ and $W_{sub}$, respectively. Second, the samples were placed in an air-tight water-filled vessel, with a pressure of 15 ± 1.5 MPa for 24 ± 2 h. After the pressurisation, the weight of the samples was again documented with $W_{pr}$. It is assumed that during this time the pressurised water fills all the capillary and most of the gel pores in the concrete, and the total porosity of the hardened concrete is the sum of suction porosity and air content. The followed procedure is based on the withdrawn Finnish standard SFS-4475: 1988 [39] that was used to measure the protective pore ratio. The air content in percentage can be calculated as follows:

(15) $$A_{air}=\frac{W_{pr}-W_{sat}}{W_{sub}}\times 100\%$$

where $W_{pr}$ is the sample weight after water pressure, $W_{sat}$ is the saturated sample weight in air, $\mathrm{and}\;W_{sub}$ is the saturated sample weight under water.

#### 4.2.3. Sample Preparation Technique for DIA

Circular discs (with the diameter of 100 mm) were sawn from the drilled cores and treated with a filler DIA. First, the samples were ground with diamond pads of grit 60 and 120 to eliminate the sawing pattern. Secondly, the pores on the surfaces were filled with blue-pigmented epoxy paste. Finally, the excess epoxy was removed by grinding with grits of 60, 120, 200, and 400, as the epoxy hardened in a few minutes. On the other hand, the grinding should remove all the epoxy that is not in the pores. The maximum grinding height was thus limited to 0.2 mm. Finally, all voids thus appear perfectly blue, while the remaining area is unaltered (i.e., cement paste and aggerates are clearly visible.) The quality of surface treatment is very important because any surface defect can be mistaken for an air void and thus cause a significant error. Therefore, the surface treatment procedure and examination procedure should be carefully conducted. In order to reduce the systematic error, at least three parallel discs were prepared at one time before scanning.

As the samples dried, an Epson Perfection V370 Photo flatbed scanner (Shinjuku, Tokyo, Japan) was used to digitise the surfaces. A square scanning area of 70 × 70 mm^2^ was selected to cover the circular cross-section of the samples. It was found out that 1200 dots per inch (DPI) offered an optimal amount of spatial resolution and speed during the scanning process, corresponding to 0.021 mm/pixel. The images were saved as the highest quality JPGs to ensure the fast processing, and also to reduce JPG compression artefacts.

Six replicate samples of the mixture GV01 were drilled from different locations (i.e., 0.5–0.55, 0.55–0.65, 0.65–0.80 mm) and employed to check the reproducibility of this DIA method. Values of area ratio of air voids that were more than 0.8 mm, and detected with the DIA method, are listed in Table 2. For an overview of these group data, the maximum coefficient of variation (CV) was 6.45%, which is less than 7%. In laboratory studies, it is expected to have CV less than 10%. It indicates that the DIA method exhibited an acceptable reproducibility in this study.

When compared to the thin-section analysis, this DIA method is significantly less time-consuming and less expensive. Moreover, higher region of interest (RoI) reduces sampling error, being one of the major error sources in thin-section analysis. Scanning allows areas of many magnitudes to be digitised and simultaneously, the operator does not have to select the RoI as the whole sample area can be analysed.

## 5. Results and Discussion

### 5.1. A Comparison among Different Pore Size Distributions of Air Voids

In this research, the pore size distribution of air voids only involved entrained air voids in the equivalent diameter ranges from 0.02 to 0.8 mm, which can be detected by this DIA method. The SS conversion was adopted to reconstruct 3-DSD of air voids. The SS conversion d is a non-parametric approach to reconstruct a sphere from a circle, and it does not assume an analytical function of size distribution. Thus, 3-DSD reconstruction operates directly on the collected 2D data.

Generally, all we need to characterise a pore size distribution curve are the mean diameter, the standard deviation, and the total number of particles per unit volume. Thus, numerical indices find much greater usefulness than the distribution curve [40]. The mean diameter is a calculated average value to characterise the property of distribution. There are many ways or formulae to express an average value of the size distribution, e.g., number-weight mean (D[1,0]), surface-weighted (Sauter) mean (D[3,2]), and volume-weighted mean (D[4,3])). In this study, the Sauter mean diameter concept was employed to describe the air voids distribution curve, owing to it having been recently used for determination of the average size of gas bubbles and particles [41]. It can be calculated according to the equation:

(16) $$d_{mean}=\frac{\sum_{i=1}^{N}N\left(i\right)d{\left(i\right)}^{3}}{\sum_{i=1}^{N}N\left(i\right)d{\left(i\right)}^{2}}$$

where N(i) and d(i) are the number and diameter of objects in the i_th class, respectively. In addition, the mode diameter is defined as the most commonly occurring size in the distribution, or it may be easier to visualise it as the highest peak seen in the distribution. The mode is not as commonly used but can be descriptive. Moreover, the median diameter is the value separating the higher half of the data from the lower half. It is easy to find the point where the cumulative distribution curve intersects the horizonal axis at the y-axis value of 0.5 (Probability). This value is one of the easier statistics to understand and one of the most meaningful for pore/particle size distributions.

**Figure 3:**
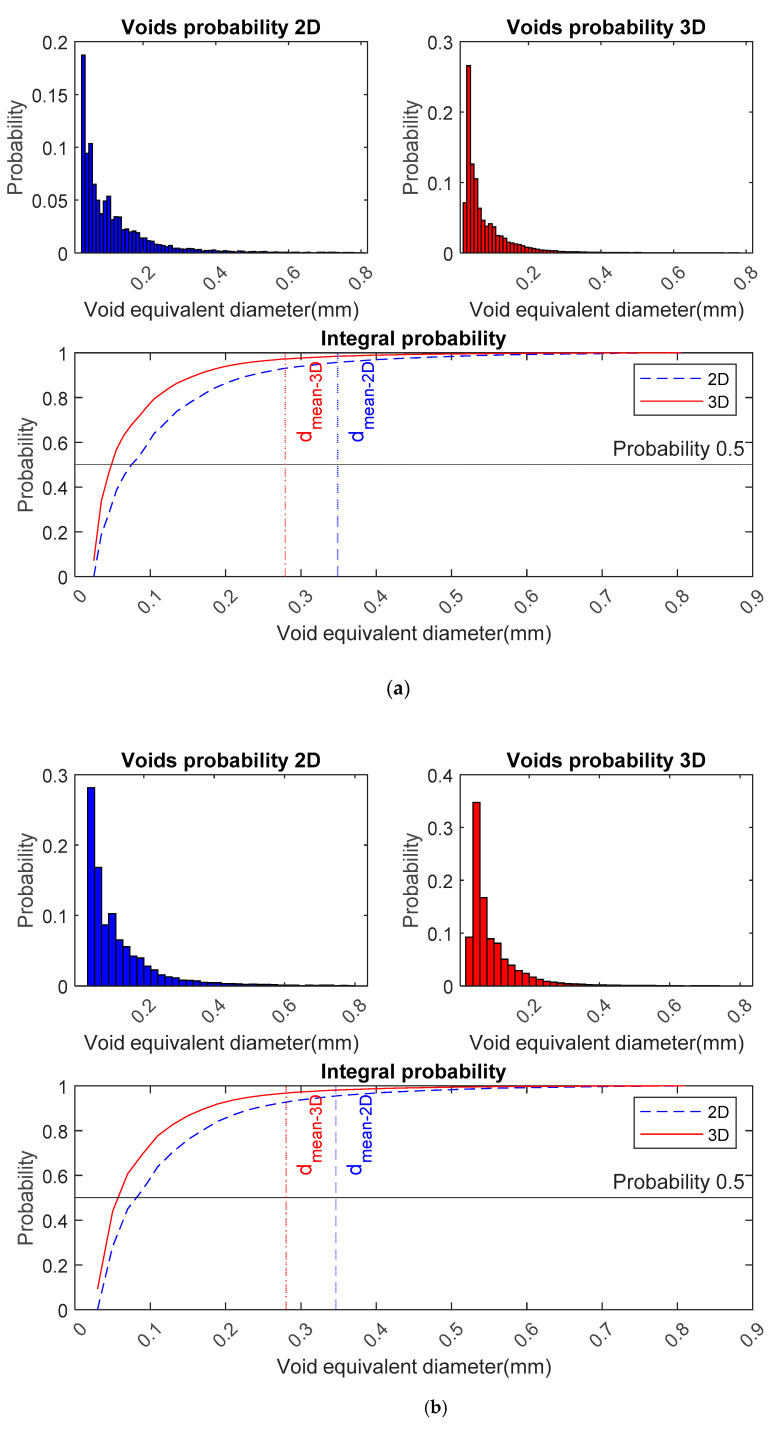

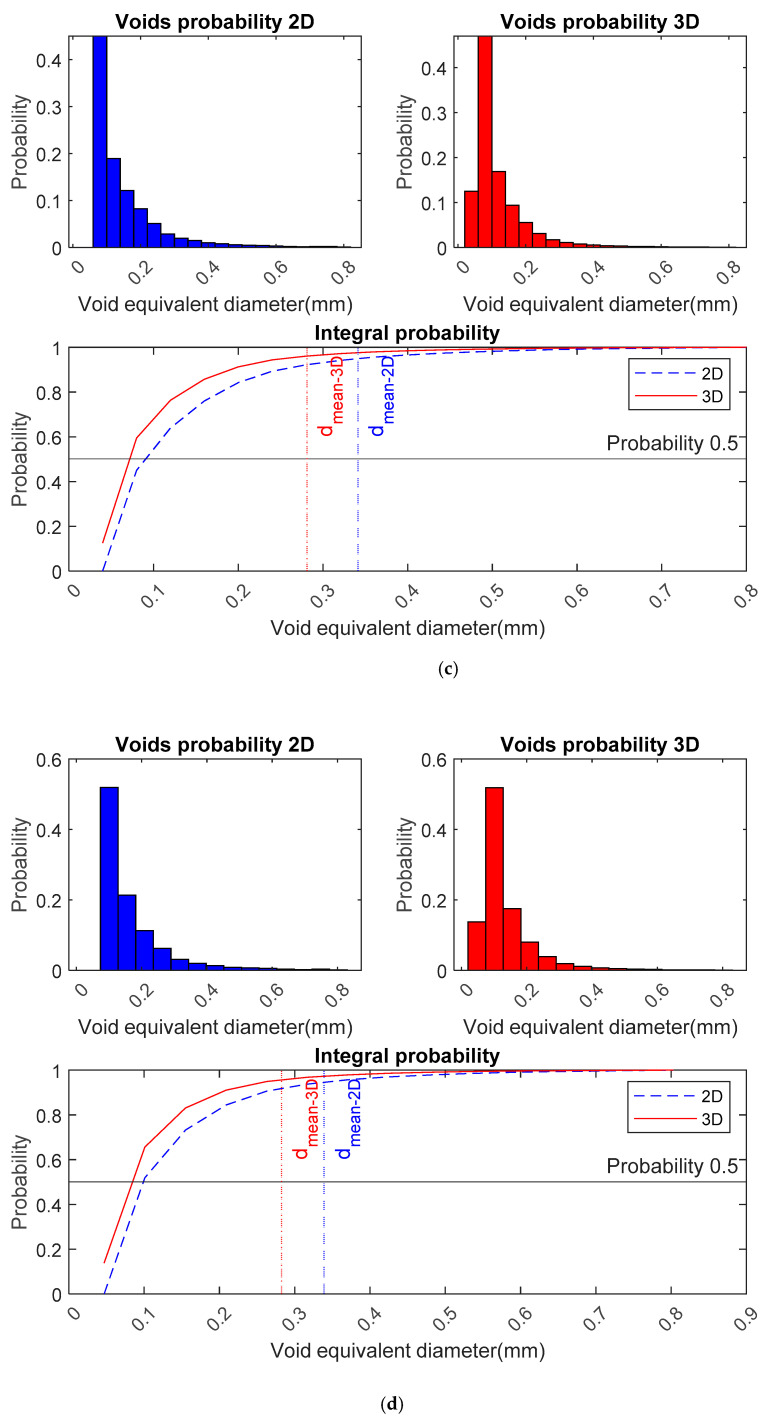
Probability distribution of air entrained voids with different class widths from 2D (blue) and 3D (red). (**a**) Class width (Δ) of 0.01 mm with 79 bins (k). (**b**) Class width (Δ) of 0.02 mm with 40 bins (k). (**c**) Class width (Δ) of 0.04 mm with 20 bins (k). (**d**) Class width (Δ) of 0.054 mm with 15 bins (k).

The reconstructed characteristic results about voids distribution with different class widths (Δ=0.01, 0.02, 0.04, 0.054 mm, corresponding to number of class sizes k=79, 40, 20, 15) based on the same image, are summarised in Figure 3 and Table 3.

#### 5.1.1. A Comparison between Curves from 2-DSD and 3-DSD

Figure 3 presents the probability distribution of air entrained voids from 2D (blue) and 3D (red). It can be seen that the curve of 3-DSD shifts to smaller voids (shift to the left) as compared with 2-DSD; also, the 3-DSD curve shows a narrow distribution while the 2-DSD curve is much broader, which is consistent with the air voids distribution curves that Fonseca presented in 2015 [23]. Values of mean diameter and median diameter of the 3-DSD (shown as red line) are smaller than the corresponding values of the 2-DSD, regardless of the class widths, and the peak of 3-DSD falls in smaller voids, and 3-DSD of air voids shifts to a narrow size range in comparison with the 2-DSD.

The total number of voids per unit represents the voids intensity. After SS conversion, it is obvious that the voids density in 2D is less than that in 3D as Table 3 shows. Although the intensity (number of spheres in class i per unit volume) can be predicted reasonably well with SS conversion, the total number of spheres per unit volume $N_{V}$ is often underestimated against the true value [33]. The value of the estimation of $N_{V}$ was obtained from the summation of the intensities of the spheres. It was reported that it appeared useful to increase the number of classes (e.g., from 20 to 80) for extracting a virtually unbiased estimate of that parameter, and as a consequence, its relative standard error and bias are 4 and 1%, respectively [42]. In this statistic with 20 and 79 classes, the standard deviation is 0.109 and 0.036, respectively, and the corresponded relative standard error is 2.4 and 0.4%. In view of this, the standard deviation in these statistics is reasonable as compared with the literature. At the same time, it arouses the interest to discuss how the class width influences the stereological results.

#### 5.1.2. A Comparison among Curves with Different Class Widths (How the Width Influences the 3D Results)

The stereological data are slightly affected by the number of size classes (actually number of size classes depending on class width) but highly susceptible to the number of observed sections [42].

As was mentioned in Section 2, during the conversion of 2D to 3D, the distributions will be generated repeatedly by changing the values of class width Δ, thereby, several groups of histograms and integral plots are generated with class widths of 0.01, 0.02, 0.04 and 0.052 mm, corresponding to 79, 40, 20 and 16 bins (bins represent the number of class size), respectively. As shown in Figure 3a–c, the bins’ detailed features corresponding to every class width are missing with the values of class width increases. Moreover, the mean diameters of 2-DSD and 3-DSD almost remain constant, irrespective of the value of class width, while the median diameters increase with the increasing of class width. It suggests that the shape of the distribution curves of air voids remains constant, regardless of the class widths. With an increase in the value of class width, the voids volume density (the total number per unit volume mm^−3^) decreases, while the standard deviation increases. This indicates the deviation of $N_{V}$ from the true value becomes large with the increase of class width. It is consistent with the report that reducing the class width can reduce the standard deviation [41]. It was also found that the standard deviation σ of 2-DSD and 3-DSD are very close to each other when the number of class size is 15, as Saltykov recommended [43]. In fact, the number of class size is dependent on the selection of class width. As Table 3 exhibits, the total number of voids per unit volume (mm^−3^) is significantly affected by class width. It really does matter how to choose class width. Takahashi et al. [33] have stated that the $N_{V,D}$ value (number of particles per unit volume) for the monodispersed system are both over- and underestimated against the true value, depending on the value of class width. Also, they recommended the number of classes should be chosen as at least 50 or more, in order to have the proper values of the population parameter in the $N_{V}\left(j\right)-d_{V}\left(j\right)$ histogram. In conclusion, it is advisable to increase the number of classes to minimise the standard deviation in the estimation [44]. However, increasing the number of classes results in a leap of total number of voids, which will affect the estimation of air content. Additionally, Cappia et al. pointed out that one of the main drawbacks of increasing the number of classes to construct the histogram is the appearance of oscillations in the class counts [32]. In addition, the digital system used in image acquisition is limited by the pixel spatial density, which can cause discretisation of the data. Thus, the selection of the number of classes in SS conversion should take account of the standard deviation and appearance of the oscillations on the estimate, as well as the resolution limit when using the image analysis.

Regardless of the 2-DSD or 3-DSD, which are based on assumptions that pores are taken as circle pores or sphere pores, respectively, the assumption of Mercury Intrusion Porometry (MIP) measurement assumes that pores are taken as cylinders. It should be noted that all these assumptions about pores depart from the reality of the pore system, which may have pores of different sizes and shapes. The DIA method cannot obtain the true pore size distribution, as the MIP method. However, they allow horizontal comparison of relative changes in different pore systems and provide a comparative assessment of the refinements that are taking place within a given system. Thus, the pore distributions of air voids from DIA method are still meaningful. Assuming that 2-DSD or 3-DSD obtained from the DIA method can provide an efficient index of assessing the frost resistance of air-entrained concrete, further research on the relation between pore size distribution of air voids and frost resistance in air-entrained concrete is needed.

### 5.2. A Comparison among the Parameters Obtained from the DIA Method, the TS Method, and the PS Method

#### 5.2.1. A Comparison between the Parameters of 2D and 3D Obtained from the DIA Method

Parameters that are related with frost resistance of air entrained concrete, e.g., air content (A), specific surface (α), and spacing factor $\bar{L}$ calculated from 2D and 3D with different class widths based on the same image using the DIA method, are summarised in Table 4. The image was from sample GV01_60_1.

With respect to the same image of the sample, as Table 4 depicted, parameters, i.e., specific surface and spacing factor from 2D and 3D, as well as air content from 2D remained almost constant irrespective of the value of class width. Only the air content from 3D was affected significantly by class width because the air content of 3D derives from the value of $N_{V}$, which is significantly affected by the class width, as Table 4 shows.

In this DIA method, one image has been captured at 1200 dpi resolution, corresponding to 0.021 mm/pixel. Considering the resolution was adopted in this DIA method, it is probably better that the class width is chosen as at least 0.021 mm or more. Although the 50 or more number of classes (50 corresponding to the class width of 0.0156 mm in this circumstance) was recommended by Takahashi [33], taking account of the resolution of this technique, a class width of 0.026 mm was selected when computing the parameters, as compared with parameters from the thin section (TS)and pressure saturation (PS) methods.

#### 5.2.2. A Comparison between Parameters Obtained from the DIA Method and That from Traditional Methods

Samples from six concrete specimens were chosen to do paralleled testing with different experimental methods, e.g., DIA, TS, PS.

As mentioned in Section 2, in this study the air content was defined as all voids with the equivalent diameter of no more than 0.8 mm that can be detected with the DIA method. Correspondingly, the 3D results indicate the voids are no more than 0.8 mm in equivalent diameter. Moreover, results for all detected pores from the DIA are presented in Table 5 as well and compared with the results from traditional methods.

**Figure 4:**
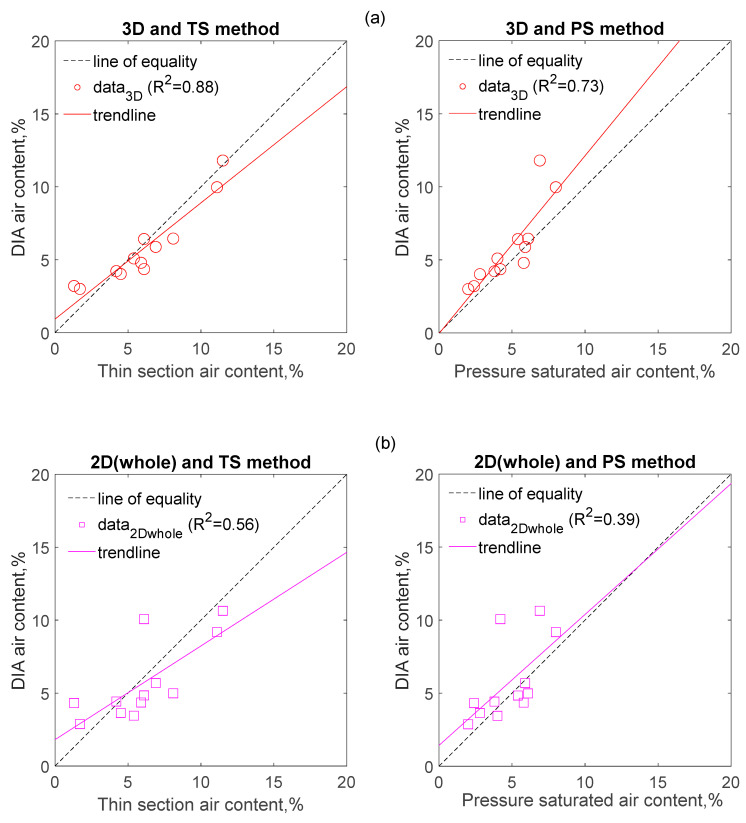

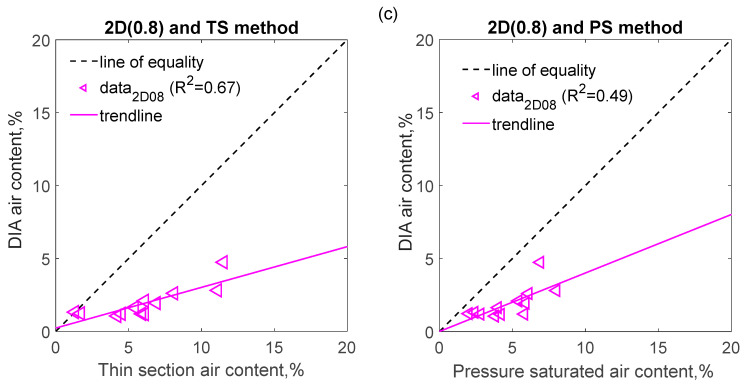
Correlation between the air content determined by DIA method and that determined by traditional methods (**a**–**c**).

**Figure 5:**
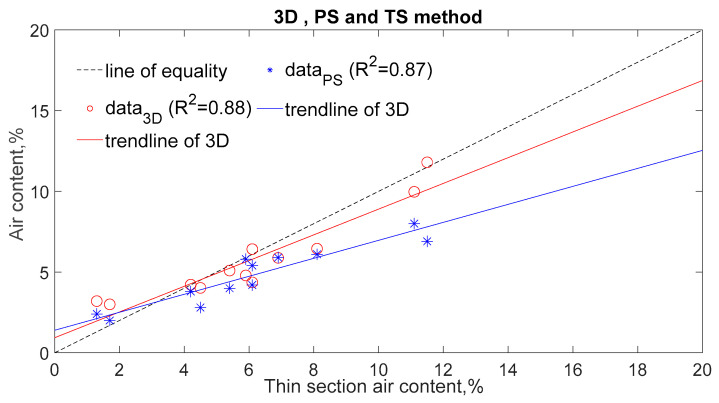
Correlations between the two different methods when air content was determined.

**Figure 6:**
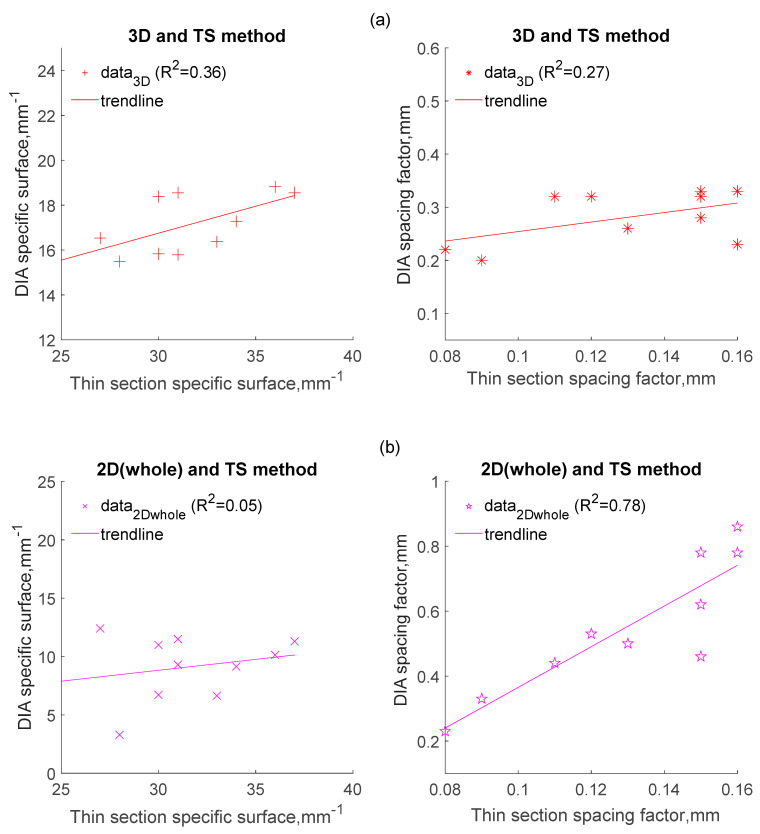

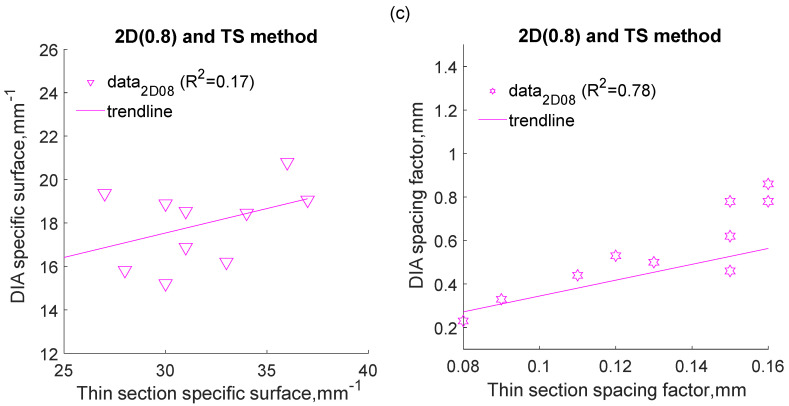
Correlation between parameters (e.g., specific surface, spacing factor) determined by DIA and that determined by thin section method (**a**–**c**).

Figure 4, Figure 5 and Figure 6 compare the parameters obtained by DIA with those obtained from the TS method as regards the air content (Figure 4 and Figure 5), and the specific surface of air voids and the spacing factor (Figure 6). The results reported in these figures were collected from 12 different samples, which were obtained from the top and bottom location of 6 different concrete specimens. The concrete specimens cover three different mixtures, with each mixture having two different vibration times.

Figure 4 indicates that the DIA method coupled with SS conversion (3D method for short) estimates the air content with more accuracy than it that without SS conversion (2D method for short). As Figure 4a presents, the air content from 3D has a better correlation with that from the TS method (correlation coefficient of 0.88), as well as air content from the PS method (correlation coefficient 0.73). As a comparison, the correlation coefficients of the whole air voids computed from 2D method with the air content from the TS method and the PS method are 0.56 and 0.39, respectively; the correlation coefficients of the air voids within 0.8 mm computed from 2D method and the air content from TS method and the PS method are 0.67 and 0.49, respectively, as shown in Figure 4b,c. In addition, it can be seen that values of air content obtained from the 2D method when taking account of air voids within 0.8 mm is smaller than that obtained from the TS and PS methods (as shown in Figure 4c).

Figure 5 shows that the correlation between air content obtained from the DIA method, and that from the TS method is as good as the correlation observed between the PS method and the TS method. It suggests the DIA method coupled with SS conversion is a logical method to estimate the air content of concrete. Despite this, a validation process should be performed on different concrete mixtures and the surface treatment procedure of samples should be kept constant before the DIA method is used.

Figure 6a–c shows that the correlation between parameters, i.e., specific surface, and spacing factor obtained from the DIA method, and those from the TS method, is not as good as the correlation observed for air content. As Figure 6b,c shows, both methods show poor correlations when the corresponding specific surfaces for the whole air voids and air voids within 0.8 are considered, respectively (correlation coefficients are 0.05 and 0.17, respectively). However, it is surprising that the DIA method shows an acceptable correlation with the thin section method when the spacing factors from 2D (without SS conversion) are considered; regardless of whole air voids or air voids within 0.8 mm, both correlation coefficients are 0.78. For the same concrete, the spacing factor is actually inversely proportional to $\overset{k}{\underset{i=1}{\sum }}N_{A}\left(i\right)d_{A}\left(i\right)$, while the air content of 2D is proportional to $\overset{k}{\underset{i=1}{\sum }}N_{A}\left(i\right)d_{A}{\left(i\right)}^{2}$. Assuming the error source is from $d_{i}$, it will have less influence on the spacing factor than the air content. As depicted in Figure 3, the values of $A_{2D}$ tend to be an underestimate as compared to the values from TS, which might reduce the error when calculating the spacing factor. Pleau et al., in 2001, gave a similar explanation based on their research [21].

It should be noted that the calculation of the spacing factor from 2D in this study is based on the same equations used in Pleau’s study. Their work on the spacing factor with image analysis has shown the values of spacing factor obtained from image analysis is very close (equal) to the values obtained from the ASTM C457 method [21]. However, this study only shows a better correlation between the spacing factor obtained from DIA and that from TS, but neither are equal. In the authors’ point of view, there are many reasons that result in the differences with the previous work. First, even though the developed thin section method is based on the ASTM C457, there are still errors between both, therefore, the same relationship cannot be expected between image analysis methods and thin section, as well as with ASTM C457. With regards to the DIA method, it is reasonable that equal values cannot be obtained when testing the spacing factor. Second, in previous work, image stitching (the field of view consists of several discrete frames in an areal analysis) was involved with regards to image analysis, therefore, frame edge effect correction [19] was considered. However, in this study, the size of individual images for image capture was 70 × 70 mm^2^ (49 cm^2^), which are representative enough without image stitching. This eliminated the effect of frame edge (boundaries of the image frame) to some extent. Thus, the frame edge effect correction of images was not considered in this research, as compared with the previous work [19,21]. Third, different resolutions were adopted in the image analysis, a pixel represents around 21 μm in this research, while a pixel represents 1.7 and 6.8 μm at magnification level of 100× and 25×, respectively, in Pleau’s research. In view of this, to correctly assess the air content of a certain concrete, how big an area needs to be analysed in the field of view when conducting the image analysis, as well as how much of the resolution is adopted, needs to be precisely defined in a standard.

After SS conversion, the air content from 3D is proportional to $\overset{k}{\underset{i=1}{\sum }}N_{V}\left(i\right)d_{V}{\left(i\right)}^{2}$, while the spacing factor is inversely proportional to $\overset{k}{\underset{i=1}{\sum }}N_{V}\left(i\right)d_{V}\left(i\right)$. It can be found from the probability distribution of air voids, as shown in Figure 3, the peaks of the 3-DSD shift to a smaller size range (the value of $d_{\begin{array}{c} mean-3D \end{array}}$ is less than $d_{mean-2D}$ ), as compared to the peaks of the 2-DSD. It suggests the air content from 3D can reach a satisfactory agreement with that from TS due to the contribution from an increase in estimation of small voids. Thus, the spacing factor from 3D will be underestimated in comparison to that from TS. It can be concluded that the estimation of air voids density, i.e., $N_{A}$, $N_{V}$ is critical when computing the characteristics of the air void system using the DIA method. Therefore, the development of a more efficient surface treatment technique and refinement of the stereological algorithm when using image analysis are still needed in further research.

## 6. Conclusions

There are several conclusions to be drawn when using the SS conversion based on the DIA method to compute the pore size distribution of air voids.

1. Values of mean diameter and median diameter of the 3-DSD of air voids are smaller than the corresponding values of the 2-DSD of air voids, regardless of the class widths. It indicates that the peak of 3-DSD of air voids falls in smaller voids, and 3-DSD of air voids shifts to a narrow size range, in comparison with the 2-DSD of air voids. In addition, it is obvious that the voids density in 2D is less than that in 3D.
2. It is found that more details of the distribution curve are missing when the value of class widths increases. The median diameters increase with the increase of class width. However, the mean diameters of 2-DSD and 3-DSD remain almost constant, irrespective of the value of class width. This suggests that the shape of the 3-DSD of air voids remains constant irrespective of the class widths.
3. With an increase in the value of class width, the voids volume density ($N_{V}$) decreases, while the standard deviation increases. This indicates the deviation of $N_{V}$ from the true value becomes large with the increasing of class width.
4. Increasing the number of classes can minimise the standard deviation in the estimation. However, it also results in a leap in the total number of voids, which will influence the estimation of air content.

Some findings to be concluded when determining the parameters of the air void system with the DIA method.

1. Parameters, i.e., specific surface and spacing factor from 2D and 3D, as well as air content from 2D, almost remained constant irrespective of the value of class width. Only the air content from 3D was affected significantly by class width because the air content of 3D derives from the value of $N_{V}$, which was significantly influenced by class width.
2. The DIA method coupled with SS conversion estimates the air content with more accuracy than without SS conversion due to the air content from 3D having a higher correlation with that from the TS method, as well as air content from the PS method, as compared with the air content from 2D. Also, the correlation between air content obtained from the DIA method and that from the TS method is as good as the correlation observed between the PS method and the TS method. It suggests the DIA method coupled with SS conversion is a logical method to estimate the air content of concrete.
3. The correlation between parameters, e.g., specific surface and spacing factor, obtained from the DIA method, and those from the TS method, are not as good as the correlation observed for the air content. However, it is surprising that the DIA method shows an acceptable correlation with the TS method when the spacing factor from 2D (without SS conversion) is considered, while both methods show poor correlations when the corresponding specific surface is considered. It can be explained that the error source is probably from $d_{i}$, because it has less influence on the spacing factor than air content.
4. The air content from 3D can reach a satisfactory agreement with that from TS due to the contribution from an increase in the estimation of small voids. Thus, it can be concluded that the estimation of air voids density, i.e., $N_{A}$, $N_{V}$ is critical when computing the characteristics of the air void system using the DIA method.

Based on this work on the air void parameters obtaining by DIA method, further studies on how these air void parameters obtained by DIA method determine freeze -thaw resistance in concrete need to be carried out.

## Figures and Tables

**Table 1:** The concrete composition of the cast concretes specimens.

Mixture Code	Water-Cement Ratio (-)	Cement (kg/m^3^)	Aggregate (kg/m^3^)	Superlasticiser (% of Cement Weight)	AEA (% of Cement Weight)	Cement Paste Volume (%)	Fresh Air Content, EN12350-7 (%)
GV01	0.44	378	1219	0.632	0.400	28.6	3.8
GV10	0.40	436	1197	0.800	0.683	30.4	6.4
GV14	0.40	430	1205	0.714	0.398	30.6	3.8

**Table 2:** Data of six replicates from mixture GV01.

Replicate No.	Area Ratio of Air Voids More than 0.8 mm (%)		
	0.5–0.55 mm	0.55–0.65 mm	0.65–0.80 mm
1	0.1342	0.2465	0.3099
2	0.1433	0.2343	0.2861
3	0.1386	0.2482	0.3223
4	0.1187	0.2739	0.2985
5	0.1271	0.2748	0.3094
6	0.1244	0.2731	0.2843
Average	0.131	0.258	0.302
STDEV	0.008	0.016	0.014
CV (%)	6.453	6.213	4.503

**Table 3:** Comparison of the characteristics for air voids distribution from 2D and 3D.

Class Width (mm)	Curve Type	Mean Diameter (mm)	Median Diameter (mm)	Total Number of Voids (mm^−2^, mm^−3^)	Standard Deviation σ
0.01	2-DSD	0.349	0.073	2.71	0.028
	3-DSD	0.279	0.044	131.5	0.036
0.02	2-DSD	0.346	0.078	2.71	0.054
	3-DSD	0.279	0.046	76.0	0.063
0.04	2-DSD	0.341	0.088	2.71	0.106
	3-DSD	0.281	0.068	42.6	0.109
0.054	2-DSD	0.339	0.096	2.71	0.137
	3-DSD	0.283	0.078	32.8	0.136

**Table 4:** Comparison of air void parameters from 2D and 3D using digital image analysis (DIA).

Class Width (mm)	Parameters from 2D			Parameters from 3D		
	A(%)	$\alpha \left(mm^{-1}\right)$	$\bar{L}\left(mm\right)$	A(%)	$\alpha \left(mm^{-1}\right)$	$\bar{L}\left(mm\right)$
0.01	4.35	6.71	0.78	6.06	15.88	0.33
0.02	4.35	6.71	0.78	4.26	15.83	0.33
0.04	4.35	6.71	0.78	3.07	15.78	0.33
0.054	4.35	6.71	0.78	2.75	15.78	0.33

**Table 5:** Comparison of air void parameters using the DIA, the TS method, and the PS method.

NO.	2D			3D			TS			PS
	A(%)	$\alpha \left(mm^{-1}\right)$	$\bar{L}\left(mm\right)$	A(%)	$\alpha \left(mm^{-1}\right)$	$\bar{L}\left(mm\right)$	A(%)	$\alpha \left(mm^{-1}\right)$	$\bar{L}\left(mm\right)$	A(%)
GV01_15_1	10.07	3.29	0.86	4.02	15.49	0.23	6.1	28	0.16	4.2
	1.18	15.82	0.59							
GV01_15_3	4.42	6.64	0.78	3.54	16.37	0.32	4.2	33	0.15	3.8
	1.06	16.20	0.60							
GV01_60_1	4.35	6.71	0.78	4.26	15.83	0.33	5.9	30	0.16	5.8
	1.20	15.21	0.61							
GV01_60_3	3.64	9.14	0.62	4.02	17.28	0.33	4.5	34	0.15	2.8
	1.20	18.46	0.50							
GV10_15_1	10.64	12.41	0.23	11.8	16.53	0.22	11.5	27	0.08	6.9
	4.74	19.36	0.27							
GV10_15_3	4.98	11.49	0.44	6.45	15.79	0.32	8.1	31	0.11	6.1
	2.61	16.88	0.40							
GV10_60_1	9.18	10.13	0.33	9.97	18.82	0.20	11.1	36	0.09	8.0
	2.81	20.79	0.31							
GV10_60_3	3.45	11.3	0.53	5.09	18.55	0.32	5.4	37	0.12	4.0
	1.64	19.06	0.44							
GV14_15_1	5.69	9.30	0.50	5.88	18.55	0.26	6.9	31	0.13	5.9
	1.94	18.54	0.42							
GV14_15_3	4.84	10.99	0.46	6.42	18.38	0.28	6.1	30	0.15	5.4
	2.10	18.89	0.40							
GV14_60_1	4.32	9.33	0.59	3.20	18.06	0.30	1.3	-	-	2.4
	1.32	20.81	0.44							
GV14_60_3	2.87	13.22	0.49	3.00	22.24	0.29	1.7	-	-	2.0
	1.23	25.21	0.38							

**Note:** Values above lines indicate the results for all voids can be detected, which was denoted with 2D (whole); values below lines indicate the results for air voids no more than 0.8 mm in equivalent diameters, which was denoted with 2D (0.8).

## Data Availability

The data presented in this study are available on request from the corresponding author.
